# Sample average treatment effect on the treated (SATT) analysis using counterfactual explanation identifies BMT and SARS-CoV-2 vaccination as protective risk factors associated with COVID-19 severity and survival in patients with multiple myeloma

**DOI:** 10.1038/s41408-023-00901-y

**Published:** 2023-12-07

**Authors:** Amit Kumar Mitra, Ujjal Kumar Mukherjee, Suman Mazumder, Vithal Madhira, Timothy Bergquist, Yu Raymond Shao, Feifan Liu, Qianqian Song, Jing Su, Shaji Kumar, Benjamin A. Bates, Noha Sharafeldin, Umit Topaloglu, National COVID Cohort Collaborative Consortium, Christopher G. Chute, Richard A. Moffitt, Melissa A. Haendel

**Affiliations:** 1 Department of Drug Discovery and Development, Harrison College of Pharmacy, Auburn University, Auburn, AL USA; 2 Center for Pharmacogenomics and Single-Cell Omics (AUPharmGx), Auburn University, Auburn, AL USA; 3 UAB Comprehensive Cancer Center, University of Alabama at Birmingham, Birmingham, AL USA; 4 Gies College of Business and Carle Illinois College of Medicine, University of Illinois, Urbana-Champaign, IL USA; 5 Palila Software LLC, Reno, NV USA; 6 Sage Bionetworks, Seattle, WA USA; 7 Duke University Medical Center, Durham, NC USA; 8 University of Massachusetts Chan Medical School, Worcester, MA USA; 9 Wake Forest School of Medicine Winston-Salem, Winston-Salem, NC USA; 10 Department of Biostatistics, Indiana University School of Medicine, Indianapolis, IN USA; 11 Division of Hematology, Department of Internal Medicine, Mayo Clinic, Rochester, MN USA; 12 Department of Medicine, Rutgers-RWJMS Medical School, New Brunswick, NJ USA; 13 School of Medicine, University of Alabama at Birmingham, Birmingham, AL USA; 14 Johns Hopkins University, Baltimore, MD USA; 15 Department of Biomedical Informatics, Stony Brook University, Stony Brook, NY USA; 16 Center for Health AI, University of Colorado School of Medicine, Aurora, CO USA

**Keywords:** Epidemiology, Myeloma

## Abstract

Patients with multiple myeloma (MM), an age-dependent neoplasm of antibody-producing plasma cells, have compromised immune systems and might be at increased risk for severe COVID-19 outcomes. This study characterizes risk factors associated with clinical indicators of COVID-19 severity and all-cause mortality in myeloma patients utilizing NCATS’ National COVID Cohort Collaborative (N3C) database. The N3C consortium is a large, centralized data resource representing the largest multi-center cohort of COVID-19 cases and controls nationwide (>16 million total patients, and >6 million confirmed COVID-19+ cases to date). Our cohort included myeloma patients (both inpatients and outpatients) within the N3C consortium who have been diagnosed with COVID-19 based on positive PCR or antigen tests or ICD-10-CM diagnosis code. The outcomes of interest include all-cause mortality (including discharge to hospice) during the index encounter and clinical indicators of severity (i.e., hospitalization/emergency department/ED visit, use of mechanical ventilation, or extracorporeal membrane oxygenation (ECMO)). Finally, causal inference analysis was performed using the Coarsened Exact Matching (CEM) and Propensity Score Matching (PSM) methods. As of 05/16/2022, the N3C consortium included 1,061,748 cancer patients, out of which 26,064 were MM patients (8,588 were COVID-19 positive). The mean age at COVID-19 diagnosis was 65.89 years, 46.8% were females, and 20.2% were of black race. 4.47% of patients died within 30 days of COVID-19 hospitalization. Overall, the survival probability was 90.7% across the course of the study. Multivariate logistic regression analysis showed histories of pulmonary and renal disease, dexamethasone, proteasome inhibitor/PI, immunomodulatory/IMiD therapies, and severe Charlson Comorbidity Index/CCI were significantly associated with higher risks of severe COVID-19 outcomes. Protective associations were observed with blood-or-marrow transplant/BMT and COVID-19 vaccination. Further, multivariate Cox proportional hazard analysis showed that high and moderate CCI levels, International Staging System (ISS) moderate or severe stage, and PI therapy were associated with worse survival, while BMT and COVID-19 vaccination were associated with lower risk of death. Finally, matched sample average treatment effect on the treated (SATT) confirmed the causal effect of BMT and vaccination status as top protective factors associated with COVID-19 risk among US patients suffering from multiple myeloma. To the best of our knowledge, this is the largest nationwide study on myeloma patients with COVID-19.

## Introduction

The coronavirus disease 19 (COVID-19) caused by Severe Acute Respiratory Syndrome Coronavirus 2 (SARS-CoV-2) has resulted in unprecedented consequences across the world in terms of mortality and quality of life [1]. Declared a pandemic by the WHO on March 11^th,^ 2020, COVID-19 has accounted for >1% of deaths globally in >180 countries, with several notable rapid surges (pandemic waves) across the world (https://covid19.who.int/) and multiple variant strains, notably B.1.1.7 (Alpha), B.1.351 (Beta), B.1.1.28 (P.1, Gamma) and B.1.617.2 (Delta) (https://www.cdc.gov/coronavirus/2019-ncov/variants/variant-classifications.html). The highly transmissible Omicron (B.1.1.529) variant that emerged in late 2021 spread in >75 countries and posed another serious threat to the already-dismal circumstances. Furthermore, multiple mutations in strain sublineages (including the most recent surge driven by the BF.7 strain of the Omicron variant in late 2022) are a serious concern owing to their ability to surpass immunity (antibody evasion) and the degree of infectivity [2].

Cancer still remains one of the major causes of death worldwide, with a rapid increase in incidence, prevalence, and mortality over the recent decades (https://seer.cancer.gov/about/). Recent studies have shown that vulnerable cancer patients infected with COVID-19 present with more severe complications compared to healthy people living in the community [3]. Furthermore, several previous studies, including ours, have reported that the risk of death is also significantly higher in cancer patients [4]. Therefore, COVID-19-related deaths in cancer patients are highly challenging, more so because of the competing and unknown risks associated with active oncologic treatment as well as with delivering patient care.

Multiple myeloma (MM) is the second-most common hematopoietic malignancy in the United States [5]. MM is an age-dependent plasma cell neoplasm characterized by clonal expansion of malignant antibody-producing post-germinal-center B cell-derived plasma cells within the bone marrow [5]. Therefore, patients with hematological malignancies, particularly multiple myeloma, have compromised immune systems due to multiple factors, including comorbidities associated with the mean age of diagnosis at ~65yo, loss of functional immunoglobulins, low CD4 + T-cell count, suppression of normal B-cell development, as well as immunosuppression through immunomodulatory drugs/IMiDs [6]. This may increase the risk of severe SARS-CoV-2/COVID-19 infection and post-acute sequelae of SARS-CoV-2/PASC/long-COVID. Moreover, myeloma patients also present with a substantial multifactorial burden of cardiovascular disease, renal impairment, lymphopenia, neutropenia, and increased risk for venous thromboembolism/VTE that may be aggravated by pre-existing conditions, disease complications, and drug toxicities which are reported as risk factors among COVID-19 patients with a potentially fatal outcome [7, 8]. In fact, an earlier study showed that myeloma patients experience 34% higher inpatient mortality due to COVID-19 [9]. Although there are a handful of studies investigating how the incidence of COVID-19, its treatment and the interaction between COVID-19 and anti-myeloma therapies affect outcomes [9, 10], there is a significant lack of studies that include substantially large datasets (>10,000 myeloma patients).

In this study, we aim to expand the previous findings on the risk factors associated with COVID-19 severity and death and the impact of anti-myeloma therapy using a very large, naturally-representative cohort of cancer patients available through the National COVID Cohort Collaborative (N3C) initiative. The NCATS’ National COVID Cohort Collaborative/N3C is a centralized data resource representing the largest multi-center cohort of COVID-19 cases and controls nationwide [11].

The NCATS’s N3C is the largest cancer cohort registry of COVID-19-tested patients nationwide that includes Electronic Health Record (EHR) data with at least one clinical encounter after January 1st, 2020 [12]. As of July 1st, 2023, N3C houses centralized data on 19,800,785 patients from 79 contributing sites. This included 7,703,019 patients who tested positive for COVID-19. Our cohort study includes 26,064 myeloma patients, out of which 8,588 were confirmed COVID-19-positive. We used this large national-level clinical registry of myeloma patients with COVID-19 to identify predisposing and treatment-related factors associated with severity and all-cause mortality within our cohort.

## Methods

### Study cohort

Our N3C myeloma cohort included patients (both inpatients and outpatients) from contributing sites who have been diagnosed with COVID-19 between January 1st, 2020, till our cut-off date May 16^th,^ 2022, 2022 (N3C release v76). All myeloma patients without COVID-19 encountered during this time period at the contributing sites were also included initially to build the overall myeloma cohort. Historical patient data from January 1st, 2018, were included for each patient from the same health system, wherever available.

### Indicator variables

The N3C clinical data set is a limited dataset that includes protected health information that may include dates of service and patient ZIP code. Details regarding data quality and harmonization checks, cohort definitions, and Malignant Neoplastic Disease standard (SNOMED) concept codes used for primary cancer diagnosis have been published earlier. Briefly, Cancer patients within the N3C registry were identified using the SNOMED Code 3633460000 by the Observational Health Data Sciences and the Informatics Atlas tool. For COVID-19 status, we used N3C positive phenotyping guidelines based on concept definitions and logic provided in Supplementary Tables 1A and 1B. For the purpose of this study, we limited our analysis to 30 days before the COVID-19 diagnosis to 30 days after the start of the index encounter. Further, we used available data to calculate indicator variables on the Charlson Comorbidity Index (CCI) adjusted for cancer diagnosis, primary cancer diagnosis, and cancer therapies.

### Myeloma diagnosis

International Staging System (ISS) for Multiple Myeloma stage was calculated using the revised guidelines provided by the International Myeloma Foundation (https://www.myeloma.org/international-staging-system-iss-reivised-iss-r-iss) as Stage 1: Alb ≥ 3.5, B2M < 3.5; Stage 2: Everything else (B2M 3.5–5.5, Albumin any); Stage 3: B2M > 5.5 [13].

### Myeloma therapies

A list of currently approved and used anti-myeloma therapies was derived from previously published clinical literature. Treatment with standard anti-myeloma chemotherapeutic regimens for each myeloma patient was assessed using a string search of each cancer therapy in the concept name and manually reviewed for correctness. Bone marrow transplantation/BMT (Hematopoietic Stem Cell Transplantation) was identified using SNOMED code 5960049, which included the vocabulary descendants of the SNOMED codes 42537745 (Bone Marrow Transplant present) and 23719005 (Transplantation of Bone Marrow).

### Severity and outcome measures

For the purpose of this myeloma patient cohort study, the outcomes of interest were: all-cause mortality (including discharge to hospice) during the index encounter, as well as clinical indicators of severity requiring hospitalization (inpatient/emergency room/intensive care unit/ICU or intensive coronary care unit/ICCU visit), or use of mechanical ventilation (N3C Procedure Concept Set ID 179437741) or extracorporeal membrane oxygenation (ECMO; N3C Procedure Concept Set ID 415149730).

### Statistical analysis and data visualization

All the analyses were performed on the Palantir platform on the N3C data enclave. Summary statistics of descriptive analyses have been represented as counts and percentages of categorical variables. The risk of severe and mild outcomes was calculated using multivariate logistic regression analysis. The models were controlled for age group, gender, race and ethnicity, smoking status, vaccination status, treatment, BMT, and CCI variables. Adjusted odds ratios were estimated with 95% Confidence intervals for potential risk factors. All tests were two-sided. Finally, Cox proportional hazard models with time to death from COVID-19 infection were used to calculate the risk of death, adjusted for age group, gender, race and ethnicity, smoking status, vaccination status, treatment, BMT, and CCI for variables. As per N3C policy, counts of <20 were not reported for privacy.

### Causal effect analysis

In this study, we performed matched sample analysis to compute the sample average treatment effect on the treated (SATT) as the measure of the causal effect of the top associated risk factors. Regression models are associative in nature and not causal. As an illustrative example, patients who did not receive BMT may have higher associated risk factors such as higher age, diabetes, high-risk cytogenetics, etc. Therefore, it is possible that patients who did not receive BMT are characterized by an inherently higher risk of mortality. While the multivariate regression models control for many covariates of significance, yet, a full causal argument is not possible due to the potential endogenous relationship between mortality risk and BMT status. The same can be stated for many other risk factors in our analysis. Therefore, it is suggested that a comparison be made across ‘matched’ samples, i.e., patients with similar characteristics other than the risk factor of interest. Accordingly, for every risk factor of interest (for example, BMT Status, Vaccination, etc.), we divided the sample into two subsamples, namely, (i) individuals with higher levels of a risk factor, and (ii) individuals with a relatively lower level of the risk factor. Examples include subsamples where individuals received BMT versus did not receive BMT, or individuals who did not receive vaccination, versus individuals who received vaccination. Note that for each risk factor, we did this subsampling separately. For each individual in the high-risk factor group, we used ‘coarsened exact matching (CEM)’ (using cem package in R, please refer to Iacus et al., 2009 [14]) to match them to individuals in the low-risk group. The matching was performed on all covariates except the risk factor of interest. For example, for the BMT status variable, we matched individuals who did not receive BMT with individuals who received BMT on all variables except BMT. In this manner, the effect of other covariates on the outcome variable (mortality) is minimized. Also, please note that in CEM, the categorical covariates are exactly matched, and the continuous covariates are approximately matched on the rough estimate of the quantiles of the continuous covariates. Therefore, each individual in the high-risk group will be matched with a small number (minimum one) of low-risk individuals on all but the risk factor of interest. Then, based on this matched sample, we computed the average difference in mortality rates between the two groups to estimate the Sample Average Treatment Effect (SATT) as explained in the paper. We also used a propensity score-based matching to check for the robustness of our results. The propensity score uses a logistics regression fit on the risk factor of interest to estimate the probability of each individual being in high or low levels of a risk factor. As an illustration, for BMT status, we first estimated a logistic regression model on all covariates to estimate the probability of an individual to receive BMT treatment. Then we grouped the patients on the propensity (probability) to receive BMT or not and compared the mortality within groups of patients with similar propensities. The results of the propensity score are very similar (not reported) to those of the CEM-matched sample analysis.

The design and development of the SATT method are non-trivial and mathematically involved. For details, please refer to Athey and Imbens 2016 [15]. Briefly, let us consider patients $i$ who received treatment $T$ (for example, BMT or vaccination). Let ${y}_{i}$ denote the response (for example, probability of death from COVID-19, referred to as “mortality or discharge to hospice”, adhering to the spirit of using sensitive language around Covid-related mortality) of patient $i$. The causal effect of the treatment is defined as the difference in the response measure under the condition that the patient received the treatment from the response measure had the patient not received the treatment. Therefore, the causal effect of the treatment on the treated ${\alpha }_{i}$ is defined as

$${\alpha }_{i}={y}_{i}\left(T=1\right)-{y}_{i}\left(T=0\right).$$

However, in observational data that is not experimentally generated, it is often not possible to observe both the response measures under treatment and no-treatment conditions. For example, for a patient in the dataset that received BMT, we only observe the response under treatment ${y}_{i}\left(T=1\right)$, but we do not observe the response under no-treatment ${y}_{i}\left(T=0\right)$. Let ${{\boldsymbol{X}}}_{i}$ denote covariates (such as patient characteristics, disease conditions, etc.) that determine the patients’ likelihood of receiving the treatment. In experimental data, treatments are usually randomized across observation units. However, in observational data, treatments are not usually randomized; rather, treatments are decided based on the covariates that determine both the treatment assignment and the response outcomes. Under the assumption that the treatment assignment is independent of the outcomes given the covariates [15], that is

$${T}_{i}\perp \left({y}_{i}\left({T}_{i}=1\right),\,{y}_{i}\left({T}_{i}=0\right)\right){\rm{|}}{{\boldsymbol{X}}}_{i}$$

It can be assumed that the response outcome of the patients in the control group can reasonably approximate the response outcome of the patients in the treatment group, given that the patients are matched on the covariates. Therefore, the treatment effect on the treated can be estimated as

$${\alpha }_{i}={y}_{i}\left(T=1\right)-{y}_{j}\left(T=0\right){\rm{|}}{X}_{i}\approx {X}_{j}.$$

The sample average treatment effect on the treated is then estimated as

$${SATT}=\frac{1}{n}\sum {\alpha }_{i}$$

Where $n$ is the number of patients who received treatment in the empirical estimation sample, we used a propensity score-based matching. First, we estimated a logistic regression model with the treatment status as the response and the covariates such as age, sex, disease stage and all other relevant variables as explanatory to predict the likelihood of patients receiving the treatment. Then we matched the treatment group with the control group patients by choosing the closest predicted likelihood of receiving the treatment. The SATT is then estimated as the sample average of the difference in the response of the treatment and the control groups.

### The role of the institutional review board

Prior institutional review board approvals were obtained from respective institutions to access the N3C data. Further, all the authors who had access to N3C data in the Enclave and performed analyses were approved by the N3C data Use Request committee to access the limited use dataset (Level 3).

## Results

**Fig. 1:**
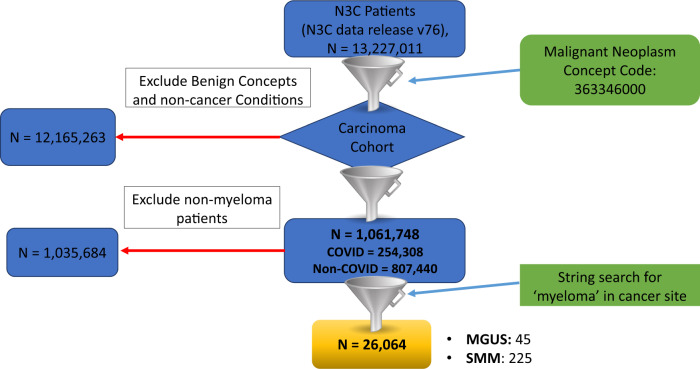
Consort diagram. Step 1: Use row-level patient data in the N3C Data Enclave, to construct a cohort of patients with myeloma. Step 2: Analysis of risk factors associated with COVID-19 severity and survival.

As of N3C data release v76 (date 05/06/2022), the N3C database consisted of 1,061,748 cancer patients, out of which 26,064 were myeloma patients (Resource Download Request ID: DRR-DCAB2E1). Among these, 8,588 myeloma patients were COVID-19 positive (Fig. 1**:** Consort diagram). In addition, 225 patients had smoldering multiple myeloma (condition: 4184985), while 45 were Monoclonal gammopathy of undetermined significance (MGUS) (conditions: 40297097, 45566693, Observations: 4149022, 37312312, 42511601). We excluded these two subgroups from our analysis. Table 1 provides detailed patient pre-admission characteristics of our study cohort, including demographic, clinical features, ISS staging, as well as COVID-19 vaccination history. According to N3C guidelines, cell sizes <20 were suppressed using a small cell size indicator (<20) to protect person privacy. To avoid cell sizes being computed from the marginal totals in cases where there is only one small cell in a row or column, we deleted the marginal total for the row or column having four or less elements and retained the small cell size indicator (<20). The mean age at diagnosis of COVID-19-positive patients was 65.89 years. 46.7% were females, and 20.2% were individuals who self-report as Black. 5.79% and 4.88% of myeloma patients were of ISS stages II and III, respectively. 25.56% had a Charlson Comorbidity Index (CCI) score of 0, 12.94% had a score of 1 and 27.54% had a score of 2 or more.

**Table 1:** Preadmission characteristics including demographic, clinical features, co-morbidities, ISS staging, and COVID-19 vaccination status.

	Multiple Myeloma	Non-COVID		COVID		Total
		17476		8588		26064
**COVID variant**			%		%	
	Alpha			2553	29.73	
	Delta			2085	24.28	
	Omicron			1487	17.31	
	Unknown			2463		
**Median age**		67.65		65.89		
**Gender**						
	FEMALE	7942	45.45	4017	46.77	11959
	MALE	9533	54.55	4570	53.21	14103
	Unknown	<20		<20		
**Age group**						
	0–17	21	0.12	<20		
	18–29	66	0.38	39	0.45	105
	30–49	1096	6.27	700	8.15	1796
	50–64	5253	30.06	2897	33.73	8150
	65+	11040	63.17	4938	57.50	15978
**Race**						
	Asian	331	1.89	177	2.06	508
	Black	4030	23.06	1739	20.25	5769
	Other	1742	9.97	1074	12.51	2816
	White	11290	64.60	5538	64.49	16828
	Unknown	83		60	0.70	
**ISS staging**						
	Stage I	5900	33.76	3023	35.20	8923
	Stage II	953	5.45	497	5.79	1450
	Stage III	1004	5.75	419	4.88	1423
	Unknown	11580		5252		
**Smoking status**						
	Current or Former	4560	26.09	2144	24.97	6704
	Non-Smoker	12878	73.69	6425	74.81	19303
	Unknown	38		<20		
**BMI**						
	Normal weight	3329	19.05	1611	18.76	4940
	Obese	4699	26.89	2243	26.12	6942
	Overweight	4228	24.19	2081	24.23	6309
	Underweight	307	1.76	139	1.62	446
	Unknown	4913		2514		
**Renal disease**	No	10826	61.95	6269	73.00	17095
	Yes	6612	37.83	2295	26.72	8907
	Unknown	38		24		
**Pulmonary disease**						
	No	12521	71.65	6867	79.96	19388
	Yes	4917	28.14	1697	19.76	6614
	Unknown	38		24	0.28	
**CCI score level**						
	Mild	4449	25.46	2194	25.55	6643
	Moderate	2907	16.63	1111	12.94	4018
	Severe	6979	39.93	2365	27.54	9344
	Unknown	3141		2918		
**Diabetes**						
	No	11233	64.28	6235	72.60	17468
	Yes	6185	35.39	2320	27.01	8505
	Unknown	58		33	0.38	
**PVD (Peripheral Vascular Disease)**						
	No	13834	79.16	7528	87.66	21362
	Yes	3584	20.51	1027	11.96	4611
	Unknown	58		33	0.38	
**COVID-19 vaccination**	No	12829	73.41	5760	67.07	18589
	Yes	4647	26.59	2828	32.93	7475
**Vaccination type**						
	JANSSEN	130	0.74	68	0.79	198
	MODERNA	1574	9.01	734	8.55	2308
	PFIZER_BIONTECH	2922	16.72	1334	15.53	4256
	OTHER	21		<20		
	NONE	12829		5760	67.07	18589

Among those with a diagnosis of COVID-19, 3.20% required invasive ventilation, and 55.73% required an inpatient or ED visit. 12.19% of patients underwent Acute kidney injury (AKI) during hospitalization (Table 2A). Overall, the survival probability was 90.7% across the course of the study. A total of 1.93% of N3C-myeloma COVID-19-positive patients died within the first 10 days, while 4.47% died in their initial 30 days of COVID-19 hospitalization (Table 2B). Table 3 provides a summary of anti-myeloma medications, including prior bone marrow transplantation. Of the patients with available data, 26.595% had a prior history of blood or marrow transplant (BMT).

**Table 2:** A: Summary of severity indicator variables with severity types. B: Summary of survival indicator variables with survival days.

A				
		Non-COVID	COVID	Total
**AKI in hospital**				
	No	14701	7536	22237
	Yes	2775	1047	3822
**Invasive ventilation**				
	No	16852	8308	25160
	Yes	624	275	899
**Inpatient or ED**				
	FALSE	5503	3778	9281
	TRUE	11935	4786	16721

B			
		Survival days	%
**COVID-19 severity_Type**			
	Unaffected	4522	52.65%
	Mild	1530	17.82%
	Mild_ED	315	3.67%
	Moderate	1570	18.28%
	Severe	77	0.90%
	Mortality or discharge to hospice	574	6.68%
**Survival days (COVID-19+ patients)**			
	0–10	166	1.93%
	11–20	137	1.60%
	21–30	81	0.94%
	31+	362	4.22%
	Died (Non COVID + )	53	0.62%
	Survived	7789	90.7%
	10-Day survival	166	98.07%
	30-Day survival	384	95.53%

**Table 3:** Medication history of COVID-19-positive myeloma patients.

		#
**Lenalidomide/Revlimid**		
	No	7154
	Yes	1410
**Bortezomib**		
	No	8020
	Yes	544
**Pomalidomide**		
	No	8079
	Yes	485
**Carfilzomib**		
	No	8362
	Yes	202
**Ixazomib**		
	No	8374
	Yes	190
**Daratumumab**		
	No	7902
	Yes	662
**Dexamethasone**	No	3358
	Yes	144
**BMT (Bone marrow transplant)**		
	No	6304
	Yes	2284

Exposure within −/+ 60 days from COVID index date.

Results of univariate analyses are shown in Table 4A. Categories from Table 1 with <20 patients were subgrouped for logistic regression analysis to obscure/suppress small cell sizes to protect person privacy. At a *p*-value threshold <0.05 and Odd ratio >1.5, prior history of hypertension (OR = 1.90; 95%CI: 1.62–2.23), PVD (OR = 1.78; 95%CI: 1.45–2.219), renal disease (OR = 2.39; 95%CI: 2.05–2.279), pulmonary disease (OR = 2.27; 95%CI: 1.92–2.68) and diabetes (OR = 2.08; 95%CI: 1.78–2.44) were significantly correlated with higher COVID-19 severity. Further, CCI score of 2 or more (OR = 2.79: 2.30–3.39) and BMI code of ‘underweight’ ( < 18.5) (OR = 2.22: 1.31–3.78) were also associated with higher levels of severity. Race (Black vs. white-p < e-99) was highly correlated with severity. On the other hand, vaccination status (OR 0.36: 0.29–0.44) and history of BMT (OR = 0.45: 0.37–0.54) showed a protective association with COVID-19 severity. Multivariate logistic regression analysis confirmed the following were found to be associated with higher rates of severity (Table 4A): history of pulmonary disease (OR 1.53) and renal disease (OR 1.54), associated with higher risks of severe outcomes. Further, a severe Charlson Comorbidity Index (CCI) score level (OR 1.78) was also associated with an increased risk of COVID-19 severity. Multivariate logistic regression analysis confirmed a negative/protective association between COVID-19 severity with BMT (Adjusted Odds Ratio or AOR 0.79) and COVID-19 vaccination (AOR 0.28). Treatment history of Dexamethasone (AOR 2.23), PI (AOR 1.5), and IMiD (AOR 1.4) therapy was found significantly correlated with an increase in the risk of COVID-19 severity.

**Table 4:** A: Multivariate logistic regression analysis results (association with severity). B: Multivariate Cox regression analysis results (association with survival).

A								
		Unadjusted analysis				Adjusted analysis		
		Odds Ratio	95% CI		*P*-value	Adjusted odds ratio	Pr(>|z | )	
	**Age**	1.033	1.03	1.04		1.031	1.09E-11	***
**Sex**	MALE	1.092	0.94	1.27	2.57E-01	0.891	2.35E-01	
**COVID_Vaccination**	Vaccinated	0.356	0.29	0.44	<2.00e-16	0.283	<2.00e-16	***
**BMI**	Obese	1.075	0.84	1.37	5.61E-01	0.801	1.52E-01	
	Overweight	1.068	0.84	1.37	6.00E-01	1.004	9.78E-01	
	Underweight	2.221	1.31	3.78	3.25E-03	1.313	4.04E-01	
**Race**	Black	1.362	0.66	2.80	4.02E-01	1.496	3.11E-01	
	Asian	0.846	0.39	1.82	6.69E-01	1.321	5.16E-01	
	Pacific_Islander	0.779	0.37	1.63	5.08E-01	1.787	1.57E-01	
**Smoking_status**	Smoker	0.858	0.73	1.02	7.42E-02	0.804	5.30E-02	.
**CCI_Level**	MODERATE	1.418	1.12	1.79	3.38E-03	1.345	6.03E-02	.
	SEVERE	2.793	2.30	3.39	<2.00e-16	1.775	9.34E-04	***
**ISS_staging**	Moderate_Severe	1.342	1.07	1.68	1.03E-02	1.516	4.14E-03	**
**Medical history**	Pulmonary	2.267	1.92	2.68	<2.00e-16	1.528	1.43E-04	***
	Hypertension	1.901	1.62	2.23	3.11E-15	0.922	4.93E-01	
	PVD	1.784	1.45	2.19	3.00E-08	1.030	8.26E-01	
	Diabetes	2.081	1.78	2.44	<2.00e-16	1.060	6.02E-01	
	Renal	2.391	2.05	2.79	<2.00e-16	1.537	3.25E-04	***
**Treatment**	IMiDs	1.046	0.87	1.25	6.28E-01	1.437	2.81E-03	**
	PI	1.000	0.80	1.26	9.98E-01	1.503	1.07E-02	*
	BMT	0.448	0.37	0.54	4.44E-16	0.790	6.68E-02	.
	Daratumumab	0.955	0.73	1.26	7.43E-01	1.090	6.37E-01	
	Dexamethasone	0.477	0.06	4.05	4.97E-01	2.231	5.78E-05	***

B						
		Adjusted Hazards Ratio (coef)	lower 0.95	upper 0.95	Pr(>|z|)	
**Age**		1.040	1.02	1.06	3.98E-07	***
**Sex**	MALE	0.793	0.57	1.09	1.58E-01	
**COVID vaccination**	Vaccinated	0.302	0.19	0.47	1.02E-07	***
**BMI**	Obese	1.045	0.67	1.64	8.48E-01	
	Overweight	1.287	0.84	1.97	2.44E-01	
	Underweight	0.808	0.28	2.31	6.90E-01	
**Race**	Black	2.630	0.36	19.21	3.41E-01	
	Asian	2.192	0.27	17.72	4.62E-01	
	Pacific_Islander	2.847	0.38	21.24	3.08E-01	
**Smoking status**	Smoker	0.918	0.65	1.30	6.27E-01	
**CCI Level**	MODERATE	3.340	1.68	6.65	6.05E-04	***
	SEVERE	5.138	2.55	10.36	4.84E-06	***
**ISS staging**	Moderate_Severe	1.549	0.98	2.45	6.22E-02	.
**Medical history**	Pulmonary	1.009	0.71	1.43	9.62E-01	
	Hypertension	0.961	0.64	1.45	8.49E-01	
	PVD	1.619	1.12	2.34	1.05E-02	*
	Diabetes	0.737	0.52	1.04	8.29E-02	.
	Renal	1.080	0.75	1.55	6.80E-01	
**Treatment**	IMiDs	1.257	0.87	1.82	2.28E-01	
	PI	1.623	1.06	2.48	2.46E-02	*
	BMT	0.653	0.42	1	5.49E-02	.
	Daratumumab	1.291	0.80	2.09	2.97E-01	

Table 4B provides a summary of multivariate Cox proportional regression analysis in the myeloma-COVID-19-positive cohort. CCI score high (Cox Hazards’ ratio/ HR 5.1; 95%CI: 2.55–10.36), CCI score moderate (HR 3.34; 95%CI: 1.68–6.65) ISS moderate or severe stage (HR 1.55; 95%CI: 0.98–2.5), history of PVD (OR 1.62; 95% 1.12–2.34) were highly significantly correlated with poorer patient survival. In addition, proteasome inhibitor treatment (HR 1.6; 95%CI: 1.1–2.5) was also significantly associated. Diabetes was correlated with better patient survival, although not significant. On the other hand, BMT (HR 0.65; 95%CI: 0.42–1) and COVID-19 vaccination (HR 0.302; 95%CI: 0.19–0.47) were associated with a significantly lower risk of death and higher survival following COVID-19 infection in myeloma patients.

Finally, we performed causal estimation using matched sample SATT method as detailed in the Methods section. A matched sample analysis follows two steps [15]. In the first step, for every individual patient in the treatment group (for example, patients who received BMT and/or vaccination), a sub-sample of patients from the control group (for example, patients who did not receive either BMT or vaccination) who are similar to the treatment group patient in every aspect other than the treatment (BMT or vaccination). The difference in the survival probability or duration (or any other relevant response measure) between the patient in the treatment group and the matched patient in the control group is the treatment effect. The average difference in the response measure between the patients in the treatment group and the control group is the SATT. Our causal effect analysis confirmed that BMT and vaccination status were associated with decreased risk of COVID-19-related death in myeloma patients, while the history of pulmonary disease, renal disease, as well as IMiD and PI therapy was significantly correlated with a high risk of death. The SATT of BMT as treatment and probability of survival status (death = 1) as the response is −0.025 (-0.031, -0.020). This indicates that MM patients who received BMT treatment are significantly less likely to die from COVID-19 as compared to those MM patients who did not receive BMT. Similarly, and not surprisingly, SATT for vaccination status is -0.123 (-0.127, -0.118) with a Welch t-Statistic of −55.186 (*p*-Value < 2.2e-16) (Supplementary Table 2). This indicates that MM patients who received vaccination are significantly less likely to die from COVID-19 than patients who did not receive the vaccination. Interestingly, the pre-existence of pulmonary and renal complications significantly increases the chances of death from COVID-19.

## Discussion

We have used the N3C patient cohort that currently includes >8 million COVID-tested patients with at least 1 clinical encounter at >75 US medical centers to construct a cohort of COVID-19 patients with multiple myeloma. To the best of our knowledge, this is the largest nationwide study on multiple myeloma patients with COVID-19 infection.

We identified several known and so-far unknown characteristics as potential risk factors for severity and death in multiple myeloma. For example, several groups, including us, have earlier established the impact of male gender and existing comorbidities as risk factors associated with COVID-19.

The impact of race on COVID-19-associated mortality/severity has been controversial. Although some studies have shown racial disparities to be significantly associated with mortality risk, several others did not find any significant effect on the rate of hospitalization or mortality. We observed significantly higher risk associated with severity in non-white ethnic groups compared to whites. These results require further in-depth analysis exploring social determinants of health, socioeconomic parameters, and access to timely and appropriate healthcare.

Furthermore, interestingly, age was not found to be significantly associated with either severity or death in our N3C-myeloma cohort. This was probably since the median age of diagnosis was already above 65 years, which has been shown as the at-risk age earlier.

We showed that vaccination with two doses of Pfizer or Moderna vaccine or a single dose of J&J vaccine showed a protective effect in the N3C-myeloma cohort. Vaccinated myeloma patients were at >350% less risk of severe outcomes and 331% less risk of death following COVID-19 infection compared to unvaccinated myeloma patients. An earlier study demonstrated that 2/3rd of vaccinated myeloma patients show some response to mRNA vaccines, although vaccination may only provide partial protection from infection, while 1/3^rd^ failed to respond based on background IgG levels of 50IU/ml [16]. However, the threshold/cut-offs were primarily probabilistic, with no clinical follow-up correlating relevant anti-spike IgG levels with protection in vaccinated patients. A recent study that measured vaccine-induced neutralizing antibodies (nAbs) in myeloma patients receiving SARS CoV-2 vaccination found that, although >80% of patients showed serological response to vaccines, several patients lack detectable virus-neutralizing activity for protection from COVID-19 which was affected by race, disease state, treatment, etc. Therefore, for a reliable evaluation of immunogenicity of COVID-19 vaccines in myeloma patients, regular management and monitoring of nAbs titer and SARS CoV-2 is crucial [17, 18].

Next, we found that bone marrow transplant (BMT) has >1.5 folds protective effect on both severity and death. On the other hand, an earlier study showed that patients with COVID-19 (including 90 patients with multiple myeloma with a prior history of autologous and allogeneic hematopoietic stem-cell transplantation (HSCT) have poor overall survival [19]). Interestingly, a recent study by Romano et al. (2022) showed that absolute monocyte count prior to SARS-CoV-2 infection is predictive of the risk of overall survival in patients with heme malignancies [20].

Next, we focused on drug classes commonly used as anti-myeloma therapies. Proteasome inhibitors (PIs) are standard-of-care/primary chemotherapeutic agents for myeloma [21–25]. Bortezomib (Bz/Velcade) was the first proteasome inhibitor to be approved by the US Food and Drug Administration (FDA) for clinical application in 2003 for the treatment of relapsed and refractory myeloma [5, 26, 27]. Other FDA-approved second-generation proteasome inhibitors used as anti-myeloma drugs include carfilzomib (Cz/Kyprolis) and the oral medication ixazomib (Ix /Ninlaro/MLN9708) [26–28]. PIs are effective anti-MM drugs when used alone or in combination with other anti-cancer agents like immunomodulatory drugs (IMiDs), alkylating agents, topoisomerase inhibitors, corticosteroids, and histone deacetylase inhibitors (HDACis) [5, 22]. More recent improvements in anti-myeloma therapeutic regimens include the monoclonal antibody Daratumumab (targeting CD-38) and chimeric antigen receptor or CAR-T-cell therapy.

Very interestingly, both our univariate and multivariate analysis showed that treatment with Immunomodulatory agents (Lenalidomide, Revlimid and Pomalidomide) was significantly associated with severe outcomes and all-cause mortality. The risk of severe outcomes was two-fold in IMiDs, while the risk of death was >2.5 folds in myeloma patients on IMiDs compared to the patients who were not on IMiDs during the study period.

Finally, anti-myeloma monoclonal antibody therapy was found to be highly protective in COVID-19-affected myeloma patients. The risk of severity was 50% lower in patients treated with daratumumab compared to patients being administered other ant-myeloma therapies. The correlation between daratumumab and IMiD-based systemic therapy, resultant immunoparesis or compromised immune system, and severe/adverse COVID-19 outcomes have so far been conflicting [29, 30]. However, most of these studies were not powered enough. Therefore, we suggest careful clinical monitoring and treatment for the management of myeloma patients with COVID-19 for immune system dysregulation during disease progression and/or immunomodulatory therapies.

Since causal effect analysis models demonstrate the ‘cause’ from a statistical standpoint, the determination of the actual biological causes needs further clinical research that compares each of these risk factors. Furthermore, a biomarker analysis, including characterization of immune and inflammatory cell populations as well as pro-inflammatory cytokines in patients with MM, will be necessary.

Nevertheless, our analysis method may serve as a template for identifying major risk factors associated with death and severity in future pandemic scenarios using large-scale patient-centered databases.

We have earlier elaborated on the strengths of the N3C database, its comparability with the manually extracted registry data from the CCC19 cohort, as well as our mechanisms to perform strict data QC, as well as the weaknesses related to the heterogeneity in data collection and reporting processes at various hospital systems, data portability, and data missingness [12]. With progressive changes within the N3C cohort and the development of more and better quality tools to extract and harmonize data, we have been able to create a robust dataset of COVID-19 patients, and non-COVID-19 controls in our database.

Overall, through the creation of the N3C-myeloma dataset, the largest COVID-19 and multiple myeloma cohort in the United States reported so far, this article summarizes the risk of severe outcomes and death/all-cause mortality associated with COVID-19 patients in multiple myeloma. Our cohort provides us with several options to perform large-scale observational studies comparing various vaccination schedules, as well as differences between severity and survival between COVID-19 variants of concern, like delta vs. Omicron.

### Supplementary information

Supplementary Tables
