# Supplementary material for: Sample average treatment effect on the treated (SATT) analysis using counterfactual explanation identifies BMT and SARS-CoV-2 vaccination as protective risk factors associated with COVID-19 severity and survival in patients with multiple myeloma
Source: Blood Cancer J. 2023 Dec 7;13(1):180. doi: 10.1038/s41408-023-00901-y (PMC10700604; doi:10.1038/s41408-023-00901-y)
Supplement: Supplementary file 1 — Supplementary Tables [file 41408_2023_901_MOESM1_ESM.docx]

**Supplementary Table 1A: Key variables and concept definitions**

| **Variable** |  | | **Concepts and Logic** |
| --- | --- | --- | --- |
|  | | ***Key Outcome*** | |
| ***COVID-19 definition*** |  | | **Concept Set Names (latest codeset ID) with Description:**  N3C Covid diagnosis (35486128)  *Description: Corresponds to ICD10CM Code: U07.1 (condition). Includes 1 Concept (Disease caused by 2019-nCoV - Concept Id: 840539006 SNOMED).*  ATLAS SARS-CoV-2 rt-PCR and AG (651620200)  *Description: Includes 55 Concepts (Measurements), characterizing a positive PCR or Antigen test.*  Atlas #818 [N3C] CovidAntibody retry (45478367)  *Description: Includes 24 Concepts (Measurements), characterizing a positive Antibody test.*  ResultsPos (400691529)  *Description: Includes 6 Concepts , collecting all the affirmative measurement results*  **Logic:**  Patient record must be associated with a “N3C Covid diagnosis”  OR  have at least one of the covid measurement concepts (“ATLAS SARS-CoV-2 rt-PCR and AG” or “[N3C] CovidAntibody retry”) AND a positive result (“ResultsPos”) |
|  | | ***Key Exposures*** | |
| Carcinoma |  | | 45029076 Neoplasm_ODT  **Logic:**  Patients with malignant neoplasm. |
| Multiple Myeloma (MM) |  | | **Logic:**  Any patient with cancer site like '%myeloma%' |
| smoldering multiple myeloma |  | | **Logic:**  Any carcinoma patient with condition concept ID 4184985 |
| Monoclonal gammopathy of undetermined significance |  | | **Logic:**  Any carcinoma patient with condition concept ID 40297097, 45566693  Any carcinoma patient with observation concept ID 4149022, 37312312, 42511601 |
|  | | ***COVID-19 Disease Severity*** | |
| Emergency Department visit |  | | **Visit Concept IDs and Names:**  262 Emergency Room and Inpatient Visit  9203 Emergency Room Visit  **Logic:**  No concept sets were used in this definition.  Includes patients with a visit start date between 14 days prior to the earliest covid diagnosis to 45 days after covid diagnosis that are also associated with one of the listed visit concept IDs. |
| Hospitalization |  | | **Visit Concept IDs and Names:**  262 Emergency Room and Inpatient Visit  8717 Inpatient Hospital  9201 Inpatient Visit  581379 Inpatient Critical Care Facility  **Logic:**  No concept sets were used in this definition. Includes patients with a visit start date between 14 days prior to the earliest covid diagnosis to 45 days after covid diagnosis that are also associated with one of the listed visit concept IDs. |
| Invasive ventilation |  | | **Concept Set Names (latest codeset ID):**  Invasive Mechanical Ventilation 2OCT20 (179437741)  **Logic:**  Includes patients associated with any procedure codes in the concept sets listed that had a procedure date on or after the earliest covid diagnosis. |
| ECMO |  | | **Concept Set Names (latest codeset ID):**  Kostka - ECMO (415149730)  **Logic:**  Includes patients associated with any procedure codes in the concept sets listed that had a procedure date on or after the earliest covid diagnosis. |
| Death |  | | **Logic:**  No concepts or concept sets were used.  Patients were flagged as deceased if a valid entry with a date was included in the death table, which is part of the OMOP data model used in the Enclave. |
|  | | ***Comorbid Conditions*** | |
| Severe cardiovascular event (Congestive heart failure and myocardial infarction) |  | | **Concept Set Names (latest codeset ID):**  Charlson - CHF (359043664)  Charlson - MI (259495957)  **Logic:**  Includes patients associated with any condition codes in the concept sets listed with an occurrence date before the earliest covid diagnosis. |
| Peripheral vascular diseases |  | | **Concept Set Names (latest codeset ID):**  Charlson - PVD (376881697)  **Logic:**  Includes patients associated with any condition codes in the concept sets listed with an occurrence date before the earliest covid diagnosis. |
| Stroke |  | | **Concept Set Names (latest codeset ID):**  Charlson - Stroke (652711186)  **Logic:**  Includes patients associated with any condition codes in the concept sets listed with an occurrence date before the earliest covid diagnosis. |
| Dementia |  | | **Concept Set Names (latest codeset ID):**  Charlson - Dementia (78746470)  **Logic:**  Includes patients associated with any condition codes in the concept sets listed with an occurrence date before the earliest covid diagnosis. |
| Pulmonary Diseases |  | | **Concept Set Names (latest codeset ID):**  Charlson - Pulmonary (514953976)  **Logic:**  Includes patients associated with any condition codes in the concept sets listed with an occurrence date before the earliest covid diagnosis. |
| Rheumatic Diseases |  | | **Concept Set Names (latest codeset ID):**  Charlson - Rheumatic (765004404)  **Logic:**  For all RA patients this value is set to 1. |
| Peptic ulcer diseases |  | | **Concept Set Names (latest codeset ID):**  Charlson - PUD (510748896)  **Logic:**  Includes patients associated with any condition codes in the concept sets listed with an occurrence date before the earliest covid diagnosis. |
| Liver diseases (mild and severe liver diseases) |  | | **Concept Set Names (latest codeset ID):**  Charlson - LiverMild (494981955)  Charlson - LiverSevere (248333963)  **Logic:**  Includes patients associated with any condition codes in the concept sets listed with an occurrence date before the earliest covid diagnosis. |
| Diabetes mellitus (diabetes mellitus and diabetes mellitus with complications) |  | | **Concept Set Names (latest codeset ID):**  Charlson - DM (719585646)  Charlson - DMcx (403438288)  **Logic:**  Includes patients associated with any condition codes in the concept sets listed with an occurrence date before the earliest covid diagnosis. |
| Renal diseases |  | | **Concept Set Names (latest codeset ID):**  Charlson - Renal (220495690)  **Logic:**  Includes patients associated with any condition codes in the concept sets listed with an occurrence date before the earliest covid diagnosis. |
| Cancer (metastatic and non-metastatic) |  | | **Concept Set Names (latest codeset ID):**  Charlson - Cancer (535274723)  Charlson - Mets (378462283)  **Logic:**  Includes patients associated with any condition codes in the concept sets listed with an occurrence date before the earliest covid diagnosis. |
|  |  | | ***COVID-19 Risk factors not included in Deyo-Charlson*** |
| Hypertension |  | | **Concept Set Names (latest codeset ID):**  [LEGEND]Hypertension - 146797511 |
| Coronary artery disease |  | | **Concept Set Names (latest codeset ID):**  Coronary Artery DiseaseV2 Atlas 811 - 630858234 |
|  |  | |  |
|  |  | | ***MM Medications*** |
| tocilizumab |  | | **Logic:**  Includes patients associated with any drug concept IDs where concept name like ‘%tocilizumab%’ |
| ibrutinib |  | | **Logic:**  Includes patients associated with any drug concept IDs where concept name like ‘%ibrutinib%’ |
| lenalidomide |  | | **Logic:**  Includes patients associated with any drug concept IDs where concept name like ‘%lenalidomide%’ |
| revlimid |  | | **Logic:**  Includes patients associated with any drug concept IDs where concept name like ‘%revlimid%’ |
| Pomalidomide |  | | **Logic:**  Includes patients associated with any drug concept IDs where concept name like ‘%pomalidomide%’ |
| Bortezomib |  | | **Logic:**  Includes patients associated with any drug concept IDs where concept name like ‘%bortezomib%’ |
| Carfilzomib |  | | **Logic:**  Includes patients associated with any drug concept IDs where concept name like ‘%carfilzomib%’ |
| Ixazomib |  | | **Logic:**  Includes patients associated with any drug concept IDs where concept name like ‘%ixazomib%’ |
| Daratumumab |  | | **Logic:**  Includes patients associated with any drug concept IDs where concept name like ‘%daratumumab%’ |
| Selinexor |  | | **Logic:**  Includes patients associated with any drug concept IDs where concept name like ‘%selinexor%’ |
| Panobinostat |  | | **Logic:**  Includes patients associated with any drug concept IDs where concept name like ‘%panobinostat%’ |
|  |  | | ***COVID Medications*** |
|  |  | | **Concept Set Names (latest codeset ID):**  Amiodarone gtt 477011432  Anakinra 614076948  Azithromycin 359938251  Chloroquine 818210864  Dexamethasone 213873961  dialysis CRRT/HD 62297511  Dobutamine gtt 41638290  Dopamine gtt 89980583  Epinephrine gtt 138921458  Epoprostenol 820810867  Esmolol gtt 437105398  Hydrocortisone 687120559  Hydroxychloroquine 726349556  Inhaled Nitric Oxide 285273342  intravenous immunoglobulin 241604784  Isoproterenol gtt 514747904  Levosimendan gtt 220815705  Lopinavir 435362039  Lopinavir/Ritonavir combination 165611849  Methylprednisolone 302593795  Milrinone gtt 316286704  Norepinephrine gtt 9512899  Phenylephrine gtt 226615355  Plasma 45129226  Prednisolone 804783116  Prednisone 520650412  Remdesivir 719693192  Ritonavir 407316475  Tocilizumab 889879486  Vasopressin gtt 308291386 |

**Supplementary Table 1B.** COVID-19 positive concepts.

| **Concept Name** | **Concept Code** | **Concept Id** | **Domain Id** | **Vocabulary Id** | **Concept Class Id** |
| --- | --- | --- | --- | --- | --- |
| COVID-19 | 840539006 | 37311061 | Condition | SNOMED | Clinical Finding |
| SARS-CoV-2 (COVID-19) N gene [Presence] in Respiratory specimen by Nucleic acid amplification using CDC primer-probe set N2 | 94757-2 | 586525 | Measurement | LOINC | Lab Test |
| SARS-related coronavirus RNA [Presence] in Specimen by NAA with probe detection | 94647-5 | 723472 | Measurement | LOINC | Lab Test |
| SARS-CoV-2 (COVID-19) N gene [Cycle Threshold #] in Specimen by Nucleic acid amplification using CDC primer-probe set N2 | 94312-6 | 706155 | Measurement | LOINC | Lab Test |
| SARS-CoV-2 (COVID-19) S gene [Cycle Threshold #] in Specimen by NAA with probe detection | 94643-4 | 723468 | Measurement | LOINC | Lab Test |
| SARS-CoV-2 (COVID-19) N gene [#/volume] (viral load) in Respiratory specimen by NAA with probe detection | 95521-1 | 36661370 | Measurement | LOINC | Lab Test |
| SARS-CoV-2 (COVID-19) S gene [Cycle Threshold #] in Respiratory specimen by NAA with probe detection | 94642-6 | 723467 | Measurement | LOINC | Lab Test |
| SARS-CoV-2 (COVID-19) N gene [Presence] in Serum or Plasma by NAA with probe detection | 94766-3 | 586520 | Measurement | LOINC | Lab Test |
| SARS-CoV-2 (COVID-19) S gene [Presence] in Respiratory specimen by NAA with probe detection | 94640-0 | 723465 | Measurement | LOINC | Lab Test |
| SARS-CoV-2 (COVID-19) [Presence] in Specimen by Organism specific culture | 94763-0 | 586516 | Measurement | LOINC | Lab Test |
| SARS-CoV-2 (COVID-19) N gene [Cycle Threshold #] in Specimen by NAA with probe detection | 94510-5 | 706167 | Measurement | LOINC | Lab Test |
| SARS-CoV-2 (COVID-19) Ag [Presence] in Respiratory specimen by Rapid immunoassay | 94558-4 | 723477 | Measurement | LOINC | Lab Test |
| SARS-CoV-2 (COVID-19) RNA [Log #/volume] (viral load) in Specimen by NAA with probe detection | 94819-0 | 715262 | Measurement | LOINC | Lab Test |
| SARS-related coronavirus N gene [Cycle Threshold #] in Specimen by Nucleic acid amplification using CDC primer-probe set N3 | 94313-4 | 706172 | Measurement | LOINC | Lab Test |
| SARS-CoV-2 (COVID-19) RNA [Presence] in Saliva (oral fluid) by NAA with probe detection | 94845-5 | 715260 | Measurement | LOINC | Lab Test |
| SARS-CoV-2 (COVID-19) S gene [Presence] in Serum or Plasma by NAA with probe detection | 94767-1 | 586519 | Measurement | LOINC | Lab Test |
| SARS-CoV-2 (COVID-19) ORF1ab region [Cycle Threshold #] in Respiratory specimen by NAA with probe detection | 94644-2 | 723469 | Measurement | LOINC | Lab Test |
| SARS-CoV-2 (COVID-19) RNA [Cycle Threshold #] in Specimen by NAA with probe detection | 94746-5 | 586529 | Measurement | LOINC | Lab Test |
| SARS-related coronavirus E gene [Presence] in Respiratory specimen by NAA with probe detection | 94758-0 | 586523 | Measurement | LOINC | Lab Test |
| SARS-CoV-2 (COVID-19) ORF1ab region [Presence] in Saliva (oral fluid) by NAA with probe detection | 95824-9 | 36031506 | Measurement | LOINC | Lab Test |
| SARS-CoV-2 (COVID-19) S gene [Presence] in Specimen by NAA with probe detection | 94641-8 | 723466 | Measurement | LOINC | Lab Test |
| SARS-CoV-2 (COVID-19) RNA [Presence] in Nasopharynx by NAA with non-probe detection | 94565-9 | 723476 | Measurement | LOINC | Lab Test |
| SARS-CoV-2 (COVID-19) N gene [Presence] in Saliva (oral fluid) by Nucleic acid amplification using CDC primer-probe set N1 | 96448-6 | 36032258 | Measurement | LOINC | Lab Test |
| SARS-CoV-2 (COVID-19) RNA [Presence] in Nasopharynx by NAA with probe detection | 94759-8 | 586526 | Measurement | LOINC | Lab Test |
| SARS-related coronavirus E gene [Presence] in Serum or Plasma by NAA with probe detection | 94765-5 | 586518 | Measurement | LOINC | Lab Test |
| SARS-CoV-2 (COVID-19) S gene [Presence] in Respiratory specimen by Sequencing | 95609-4 | 36031213 | Measurement | LOINC | Lab Test |
| SARS-CoV-2 (COVID-19) RNA [Presence] in Nose by NAA with probe detection | 95406-5 | 757677 | Measurement | LOINC | Lab Test |
| SARS-CoV-2 (COVID-19) N gene [Presence] in Specimen by Nucleic acid amplification using CDC primer-probe set N2 | 94308-4 | 706154 | Measurement | LOINC | Lab Test |
| SARS-CoV-2 (COVID-19) RNA panel - Respiratory specimen by NAA with probe detection | 94531-1 | 706158 | Measurement | LOINC | Lab Test |
| SARS-CoV-2 (COVID-19) N gene [Presence] in Respiratory specimen by NAA with probe detection | 94533-7 | 706161 | Measurement | LOINC | Lab Test |
| SARS-CoV-2 (COVID-19) RdRp gene [Cycle Threshold #] in Specimen by NAA with probe detection | 94645-9 | 723470 | Measurement | LOINC | Lab Test |
| SARS-CoV-2 (COVID-19) RdRp gene [Presence] in Lower respiratory specimen by NAA with probe detection | 96120-1 | 36031652 | Measurement | LOINC | Lab Test |
| SARS-CoV-2 (COVID-19) N gene [Presence] in Saliva (oral fluid) by NAA with probe detection | 95425-5 | 36661378 | Measurement | LOINC | Lab Test |
| SARS-related coronavirus+MERS coronavirus RNA [Presence] in Respiratory specimen by NAA with probe detection | 94532-9 | 706159 | Measurement | LOINC | Lab Test |
| SARS-related coronavirus E gene [Presence] in Specimen by NAA with probe detection | 94315-9 | 706174 | Measurement | LOINC | Lab Test |
| SARS-CoV-2 (COVID-19) N gene [Presence] in Specimen by Nucleic acid amplification using CDC primer-probe set N1 | 94307-6 | 706156 | Measurement | LOINC | Lab Test |
| SARS-CoV-2 (COVID-19) RNA [Cycle Threshold #] in Respiratory specimen by NAA with probe detection | 94745-7 | 586528 | Measurement | LOINC | Lab Test |
| Measurement of Severe acute respiratory syndrome coronavirus 2 antigen | 1240471000000102 | 37310257 | Measurement | SNOMED | Observable Entity |
| SARS-related coronavirus E gene [Cycle Threshold #] in Specimen by NAA with probe detection | 94509-7 | 706166 | Measurement | LOINC | Lab Test |
| SARS-CoV-2 (COVID-19) Ag [Presence] in Upper respiratory specimen by Immunoassay | 96119-3 | 36032419 | Measurement | LOINC | Lab Test |
| SARS-CoV-2 (COVID-19) RNA panel - Specimen by NAA with probe detection | 94306-8 | 706169 | Measurement | LOINC | Lab Test |
| SARS-CoV-2 (COVID-19) RNA [Presence] in Respiratory specimen by NAA with non-probe detection | 95608-6 | 36031238 | Measurement | LOINC | Lab Test |
| SARS-CoV-2 (COVID-19) RdRp gene [Presence] in Respiratory specimen by NAA with probe detection | 94534-5 | 706160 | Measurement | LOINC | Lab Test |
| SARS-CoV-2 (COVID-19) N gene [Presence] in Nasopharynx by NAA with probe detection | 94760-6 | 715272 | Measurement | LOINC | Lab Test |
| SARS-CoV-2 (COVID-19) N gene [Presence] in Nose by NAA with probe detection | 95409-9 | 757678 | Measurement | LOINC | Lab Test |
| SARS-CoV-2 (COVID-19) RNA [Presence] in Saliva (oral fluid) by Sequencing | 94822-4 | 715261 | Measurement | LOINC | Lab Test |
| SARS-CoV-2 (COVID-19) RNA [Presence] in Specimen by NAA with probe detection | 94309-2 | 706170 | Measurement | LOINC | Lab Test |
| SARS-CoV-2 (COVID-19) N gene [Cycle Threshold #] in Specimen by Nucleic acid amplification using CDC primer-probe set N1 | 94311-8 | 706157 | Measurement | LOINC | Lab Test |
| SARS-CoV-2 (COVID-19) ORF1ab region [Presence] in Respiratory specimen by NAA with probe detection | 94559-2 | 723478 | Measurement | LOINC | Lab Test |
| SARS-related coronavirus N gene [Presence] in Specimen by Nucleic acid amplification using CDC primer-probe set N3 | 94310-0 | 706171 | Measurement | LOINC | Lab Test |
| SARS-CoV+SARS-CoV-2 (COVID-19) Ag [Presence] in Respiratory specimen by Rapid immunoassay | 95209-3 | 757685 | Measurement | LOINC | Lab Test |
| SARS-CoV-2 (COVID-19) RNA [Presence] in Respiratory specimen by Sequencing | 95424-8 | 36661377 | Measurement | LOINC | Lab Test |
| SARS-CoV-2 (COVID-19) N gene [Log #/volume] (viral load) in Respiratory specimen by NAA with probe detection | 95522-9 | 36661371 | Measurement | LOINC | Lab Test |
| SARS-CoV-2 (COVID-19) RdRp gene [Cycle Threshold #] in Respiratory specimen by NAA with probe detection | 94646-7 | 723471 | Measurement | LOINC | Lab Test |
| SARS-CoV-2 (COVID-19) RdRp gene [Presence] in Upper respiratory specimen by NAA with probe detection | 96123-5 | 36031453 | Measurement | LOINC | Lab Test |
| SARS-CoV-2 (COVID-19) RdRp gene [Presence] in Specimen by NAA with probe detection | 94314-2 | 706173 | Measurement | LOINC | Lab Test |
| SARS-CoV-2 (COVID-19) N gene [Presence] in Specimen by NAA with probe detection | 94316-7 | 706175 | Measurement | LOINC | Lab Test |
| SARS-CoV-2 (COVID-19) ORF1ab region [Cycle Threshold #] in Specimen by NAA with probe detection | 94511-3 | 706168 | Measurement | LOINC | Lab Test |
| SARS-CoV-2 (COVID-19) N gene [Presence] in Respiratory specimen by Nucleic acid amplification using CDC primer-probe set N1 | 94756-4 | 586524 | Measurement | LOINC | Lab Test |
| SARS-CoV-2 (COVID-19) ORF1ab region [Presence] in Specimen by NAA with probe detection | 94639-2 | 723464 | Measurement | LOINC | Lab Test |
| SARS-related coronavirus RNA [Presence] in Respiratory specimen by NAA with probe detection | 94502-2 | 706165 | Measurement | LOINC | Lab Test |
| SARS-CoV-2 (COVID-19) RNA panel - Saliva (oral fluid) by NAA with probe detection | 95826-4 | 36032061 | Measurement | LOINC | Lab Test |
| SARS-CoV-2 (COVID-19) RNA [Presence] in Respiratory specimen by NAA with probe detection | 94500-6 | 706163 | Measurement | LOINC | Lab Test |
| SARS-CoV-2 (COVID-19) specific TCRB gene rearrangements [Presence] in Blood by Sequencing | 95970-0 | 36031944 | Measurement | LOINC | Lab Test |
| SARS-CoV-2 (COVID-19) RNA [Presence] in Serum or Plasma by NAA with probe detection | 94660-8 | 723463 | Measurement | LOINC | Lab Test |
| SARS-CoV-2 (COVID-19) IgG Ab [Presence] in Serum, Plasma or Blood by Rapid immunoassay | 94507-1 | 706181 | Measurement | LOINC | Lab Test |
| SARS-CoV-2 (COVID-19) IgA Ab [Units/volume] in Serum or Plasma by Immunoassay | 94720-0 | 723459 | Measurement | LOINC | Lab Test |
| SARS-CoV-2 (COVID-19) IgM Ab [Presence] in Serum, Plasma or Blood by Rapid immunoassay | 94508-9 | 706180 | Measurement | LOINC | Lab Test |
| SARS-CoV-2 (COVID-19) neutralizing antibody [Presence] in Serum by pVNT | 95411-5 | 757680 | Measurement | LOINC | Lab Test |
| SARS-CoV-2 (COVID-19) IgG+IgM Ab [Presence] in Serum or Plasma by Immunoassay | 94547-7 | 723479 | Measurement | LOINC | Lab Test |
| SARS-CoV-2 (COVID-19) Ab panel - Serum, Plasma or Blood by Rapid immunoassay | 94503-0 | 706176 | Measurement | LOINC | Lab Test |
| SARS-CoV-2 (COVID-19) IgM Ab [Units/volume] in Serum or Plasma by Immunoassay | 94506-3 | 706178 | Measurement | LOINC | Lab Test |
| SARS-CoV-2 (COVID-19) IgA Ab [Presence] in Serum or Plasma by Immunoassay | 94562-6 | 723473 | Measurement | LOINC | Lab Test |
| SARS-CoV-2 (COVID-19) neutralizing antibody [Titer] in Serum by pVNT | 95410-7 | 757679 | Measurement | LOINC | Lab Test |
| SARS-CoV-2 (COVID-19) Ab [Presence] in Serum or Plasma by Immunoassay | 94762-2 | 586515 | Measurement | LOINC | Lab Test |
| SARS-CoV-2 (COVID-19) IgG Ab [Units/volume] in Serum or Plasma by Immunoassay | 94505-5 | 706177 | Measurement | LOINC | Lab Test |
| SARS-CoV-2 (COVID-19) Ab [Units/volume] in Serum or Plasma by Immunoassay | 94769-7 | 586522 | Measurement | LOINC | Lab Test |
| SARS-CoV-2 (COVID-19) IgA+IgM [Presence] in Serum or Plasma by Immunoassay | 95125-1 | 757686 | Measurement | LOINC | Lab Test |
| SARS-CoV-2 (COVID-19) IgG Ab [Presence] in Serum or Plasma by Immunoassay | 94563-4 | 723474 | Measurement | LOINC | Lab Test |
| SARS-CoV-2 (COVID-19) Ab panel - Serum or Plasma by Immunoassay | 94504-8 | 706179 | Measurement | LOINC | Lab Test |
| SARS-CoV-2 (COVID-19) IgA Ab [Presence] in Serum, Plasma or Blood by Rapid immunoassay | 94768-9 | 586521 | Measurement | LOINC | Lab Test |
| SARS-CoV-2 (COVID-19) IgM Ab [Presence] in Serum or Plasma by Immunoassay | 94564-2 | 723475 | Measurement | LOINC | Lab Test |
| SARS-CoV-2 (COVID-19) Ab [Interpretation] in Serum or Plasma | 94661-6 | 723480 | Measurement | LOINC | Lab Test |
| SARS-CoV-2 (COVID-19) IgG Ab [Presence] in DBS by Immunoassay | 94761-4 | 586527 | Measurement | LOINC | Lab Test |
|  |  |  |  |  |  |
|  |  |  |  |  |  |
| Presumptive positive | 720735008 | 36715206 | Meas Value | SNOMED | Qualifier Value |
| Positive | 10828004 | 9191 | Meas Value | SNOMED | Qualifier Value |
| Presumptive positive | LA31317-3 | 36032716 | Meas Value | LOINC | Answer |
| Positive | LA6576-8 | 45884084 | Meas Value | LOINC | Answer |
| Detected | LA11882-0 | 45877985 | Meas Value | LOINC | Answer |
| Present | LA9633-4 | 45879438 | Meas Value | LOINC | Answer |
| Abnormal | LA12748-2 | 45878745 | Meas Value | LOINC | Answer |
| Present | 52101004 | 4181412 | Meas Value | SNOMED | Qualifier Value |
| Reactive | LA15255-5 | 45881802 | Meas Value | LOINC | Answer |
| Detected | 260373001 | 4126681 | Meas Value | SNOMED | Qualifier Value |

**Supplementary Table 2.** Top Significant results from SATT analysis showing protective/lower risks associated with BMT and COVID-19 Vaccination status.

|  | **y vs x** | **Mean death probability of y** | **Mean death probability of x** | **Difference of death probability (SATT)** | **95% confidence interval** | **Welch Two sample t-test** |  |
| --- | --- | --- | --- | --- | --- | --- | --- |
| **BMT Status** | Bmt = 1 vs Bmt = 0 | 0.131 | 0.156 | -0.025 | -0.020 to -0.031 | t = 9.5182, df = 7832.5, | p-value < 2.2e-16 |
| **COVID-19 Vaccination** | Vx_flag = 1 vs Vx_flag = 0 | 0.068 | 0.191 | -0.123 | -0.118 to -0.127 | t = 55.186, df = 5547.2 | p-value < 2.2e-16 |
| **IMiD Therapy** | IMiDs_flag = 1 vs IMiDs_flag = 0 | 0.184 | 0.141 | 0.043 | 0.049 to 0.037 | t = -14.564, df = 7705.3 | p-value < 2.2e-16 |
| **PI Therapy** | PI_flag = 1 vs PI_flag = 0 | 0.195 | 0.145 | 0.05 | 0.056 to 0.044 | t = -16.479, df = 7655.9 | p-value < 2.2e-16 |
| **Daratumumab Therapy** | Daratumumab_flag = 1 vs Daratumumab_flag = 0 | 0.159 | 0.149 | 0.01 | 0.015 to 0.004 | t = -3.4398, df = 7946.1 | p-value = 0.000585 |
| **Pulmonary disease** | Pulmonary = 1 vs Pulmonary = 0 | 0.187 | 0.136 | 0.051 | 0.056 to 0.045 | t = -17.725, df = 7600.4 | p-value < 2.2e-16 |
| **Renal Disease** | Renal = 1 vs Renal = 0 | 0.182 | 0.132 | 0.05 | 0.055 to 0.045 | t = -18.432, df = 7566.8 | p-value < 2.2e-16 |
